# Connecting the dots between cell populations, whole-brain activity, and behavior

**DOI:** 10.1117/1.NPh.9.3.032208

**Published:** 2022-03-26

**Authors:** Lauren N. Beloate, Nanyin Zhang

**Affiliations:** a Pennsylvania State University, Department of Biomedical Engineering, Pennsylvania, United States; b Pennsylvania State University, Huck Institutes of the Life Sciences, Pennsylvania, United States

**Keywords:** functional magnetic resonance imaging, optogenetics, rodent, calcium, Ca^2+^, awake, anesthetized

## Abstract

Simultaneously manipulating and monitoring both microscopic and macroscopic brain activity *in vivo* and identifying the linkage to behavior are powerful tools in neuroscience research. These capabilities have been realized with the recent technical advances of optogenetics and its combination with fMRI, here termed “opto-fMRI.” Opto-fMRI allows for targeted brain region-, cell-type-, or projection-specific manipulation and targeted ${\mathrm{Ca}}^{2+}$ activity measurement to be linked with global brain signaling and behavior. We cover the history, technical advances, applications, and important considerations of opto-fMRI in anesthetized and awake rodents and the future directions of the combined techniques in neuroscience and neuroimaging.

## 1 Introduction

Optogenetics and functional magnetic resonance imaging (fMRI) are two distinct neuroscience methods with completely different working mechanisms. fMRI maps dynamic activities across the whole brain based on neural activity-induced hemodynamic changes.^1^^–^^3^ Optogenetics is a tool, combining genetic and optical methods, that allows for the manipulation or monitoring of specific cell populations, brain regions, or neural pathways to study the effect on brain function and behavior.^4^ Combining these two technologies (i.e., opto-fMRI) has offered tremendous synergy in neuroscience research. In this article, we first briefly review the history of fMRI and optogenetics and then summarize studies exemplifying the advantages of opto-fMRI. The literature that we review here is not exclusive, and the topic has also been discussed in several other excellent review articles.^5^^–^^7^

### 1.1 Brief History of fMRI in Rodents

As opto-fMRI is typically carried out in rodents, here we briefly review the history of fMRI with a focus on rats and mice; however, fMRI provides a noninvasive way to measure brain activity in humans, nonhuman primates, rodents, and other species.^8^^–^^10^ With high-field scanners (1.5 T or higher) and variations on technical applications, blood oxygen level-dependent (BOLD) imaging allows for high spatial resolution, whole-brain coverage, and relatively quick scan times. BOLD imaging is the basis of fMRI, and it functions under the assumption that changes in the intensity of the MRI signal depend on changes in the level of blood oxygenation in the brain.^11^ This, in turn, provides an indirect measure of changes in local and global brain activity through the process of neurovascular coupling.^12^ Although the individual components can be measured in isolation, changes in the BOLD signal reflect a combination of multiple aspects of the hemodynamic and metabolic responses, including cerebral blood flow (CBF), cerebral blood volume (CBV), and cerebral metabolic rate of oxygen.^13^^,^^14^ There are many variations in the technical application of BOLD fMRI, and as with many techniques, there are trade-offs to consider when choosing the best approach. For instance, gradient echo sequences allow for very rapid image acquisition but can be vulnerable to susceptibility and/or large-vein artifacts.^15^ If more precise spatial mappings are preferred, a spin echo sequence can be employed instead.^16^ Depending on the specific scientific questions, there are also various fMRI approaches to experimental design as applied in rodents, each with its own advantages. These include task-based fMRI, resting-state fMRI (rs-fMRI), and pharmacological fMRI (phMRI).^8^^,^^17^^–^^20^ Early task-based fMRI applications allowed for the study of multiple brain systems in rodents, such as the visual, somatosensory, and motor systems.^21^^–^^23^ In addition, rs-fMRI in awake and anesthetized rodents^17^^–^^19^ has enabled the detection of functional connectivity patterns and resting-state networks similar to those seen in humans.^24^^–^^28^ phMRI has also helped elucidate the effects of different compounds on the brain in terms of activated brain sites and drug kinetics.^29^

Notably, early fMRI applications in rodents were carried out in the anesthetized state as anesthesia was needed to limit the animal’s motion during imaging. Although imaging in anesthetized rodents is undoubtedly valuable and necessary in many cases, fMRI in nonanesthetized rodent models offers the unique translational advantage of being able to validate, expand upon, and better understand imaging results in humans. Furthermore, awake rodent imaging is essential for the interpretation of correlations between neural activity/connectivity and behavior^30^^,^^31^ as the neurovascular coupling relationship and functional connectivity results do not rely on the type and dosage of anesthetics involved.^32^^–^^36^ For a comprehensive review on the technical advances of awake animal imaging and the effects of anesthesia on imaging results, see Ref. 32.

### 1.2 History of Optogenetics

The term “optogenetics” (first coined here: Ref. 37) broadly refers to any combined use of genetic targeting and optical interrogation, although it has most commonly been used to refer to the use of optical probes for the activation or inhibition of cells. Here, we divide it into two components: the optical actuators and the indicators, or sensors. For a historical review of the first decade of optogenetics, see Ref. 38.

#### 1.2.1 Genetically engineered actuators

Optogenetics has been used to elucidate the role of defined cell types and projections in both natural behaviors and those related to brain disorders in multiple animal models. With the exception of the “Future Directions” section, the current review focuses primarily on work done in rodents, but optogenetics has also been employed in drosopholia, zebrafish, and nonhuman primates.^39^^–^^41^ Optogenetics has now been utilized in experiments across countless neuroscience subfields, including feeding,^42^ arousal,^43^ mating,^44^ drug reward,^45^^,^^46^ fear,^47^ and learning and memory.^48^ Along with appropriate controls (for this direction, see Ref. 38), multiple factors need to be carefully considered when using optogenetics, including choice of opsin, viral vector, and light source. The two most commonly used families of optogenetic actuators, or opsins, are currently the inhibitory halorhodopsins (NpHR), which pump Cl- ions into the cell and cause a hyperpolarizing current,^49^ and the excitatory channelrhodopsins (ChR2), which allow positively charged ions to flow freely through the opsin pore, leading to a depolarizing current.^4^ Since the early versions of opsins, many more have been discovered and developed to allow for faster kinetics, bistable properties, altered ion conductance, shifted color-response properties, transsynaptic or conditional expression, and use of immediate early gene promoters.^50^^–^^56^ For a more complete review of viral vectors that partly allow for these advances, see Ref. 57. Avoiding the additional need for viral injection surgery, there are also genetic mouse and rat models that express various opsins.^58^ Early implementation of optogenetics utilized lasers as the primary light source; however, light-emitting diodes (LEDs) are now regularly used, and implantable LEDs that can be controlled wirelessly are also commercially available.^59^^,^^60^

Two major advantages of optogenetics include high cell-type specificity and high temporal precision. Opsins can be selectively expressed in excitatory or inhibitory neurons, astrocytes or other specialized cells. For example, in an early optogenetic study, hypocretin (Hcrt)::ChR2-mCherry lentivirus was injected into the lateral hypothalamus of Hcrt::EGFP transgenic mice to optogenetically investigate the role of hypothalamic Hcrt (also called orexin) in sleep-wake transitions.^43^ ChR2-mCherry was expressed in 88% of EGFP-expressing Hcrt cells, and expression lasted for at least 2 months.^43^ There have now been countless improvements upon viral vector technology and development of transgenic rodent models that allow for the targeted expression of opsins based on virtually any cell type, projection, and even functionality. One such advancement is a high-photocurrent ChR2 from the species *Chloromonas oogama* (CoChR) that can be fused with a segment of the kainite receptor KA2 subunit to allow for specific cell body expression: a somatic CoChR (soCoChR).^61^ The application of two-photon computer-generated holography illumination on soCoChR-expressing cells led to a <15  ms action potential latency with a <1  ms spike jitter in brain slices.^61^ These examples, along with others, illustrate that opsins can be selectively expressed in a specified cell population, and turning opsins on and off can occur within submilliseconds. Furthermore, the addition of optogenetics with other technologies extends its specificity and precision capabilities even further, sometimes to the scale of single-cell investigations. For instance, the concurrent application of two-photon optogenetic activation with a spatial light modulator and calcium imaging in the mouse barrel cortex allowed for the functionally defined, user-selected interrogation of a specific, small population of neurons across behavioral states.^62^ On the other hand, the combination of optogenetics with technologies such as fMRI allows for targeted neural manipulations while visualizing the whole-brain response.

#### 1.2.2 Genetically engineered indicators

There is a wide array of indicators of neuronal activity that take advantage of optogenetic technology, including genetically encoded calcium indicators (GECIs). Calcium (${\mathrm{Ca}}^{2+}$) influx has long been used as a proxy for neuronal activity,^63^ and ${\mathrm{Ca}}^{2+}$ activity has been correlated with behavioral tasks in rodents.^42^^,^^64^^–^^67^ There are several different types of ${\mathrm{Ca}}^{2+}$ indicators, but the most widely used GECI is GCaMP. Using a single fluorophore ${\mathrm{Ca}}^{2+}$ indicator, GCaMPs allow us to image ${\mathrm{Ca}}^{2+}$ activity with single-cell resolution while still maintaining relatively high temporal resolution.^68^ For reviews on the history and development of ${\mathrm{Ca}}^{2+}$ indicators, see Refs. 69 and 70. To image neural tissue *in vivo* from different depths of brain regions, a few techniques have been utilized in conjunction with GECIs: head-fixed single-photon^71^^–^^73^ and two-photon mesoscopic imaging,^74^ miniscopes,^75^ and fiber photometry.^76^^,^^77^ The development in technology over the past 25 years, such as the extension of the GECI color palette, has allowed for the measurement of neural activity in distinct and even spatially intermingled cell populations and all-optical, cellular resolution circuit mapping.^78^^–^^80^

Like optogenetic actuators, GCaMP can be selectively expressed in a specific cell population using similar genetic and viral methods to monitor the ${\mathrm{Ca}}^{2+}$ dynamics of a subpopulation of cells in a specified brain region. Of the GCaMPs, GCaMP6 is currently the most widely used, with versions that have been developed for sensitivity (GCaMP6s), faster kinetics (GCaMP6f), and a balance between the two (GCaMP6m).^81^ In cultured neurons, GCaMP6s displayed a threefold higher affinity for ${\mathrm{Ca}}^{2+}$ compared with its predecessor, GCaMP5G, and GCaMP6f displayed a twofold faster rise time and 1.7-fold faster decay time than GCaMP5G.^81^ Furthermore, in the *in vivo* mouse visual cortex, the fraction of responding neurons detected with GCaMP6s was threefold higher than those of GCaMP5G.^81^ Although GCaMP6m and GCaMP6f signals led to lower spike detection efficiencies, they displayed faster kinetics *in vivo*, similar to their *in vitro* results.^81^ Improvements to GCaMPs are continuously being made, and as with optogenetic actuators, ${\mathrm{Ca}}^{2+}$ imaging using optogenetic indicators can be combined with other techniques to contribute to a well-rounded story of the brain and its role in the expression of behavior. These contributions are discussed throughout the body of the current review.

### 1.3 Advantages of Opto-fMRI

**Fig. 1:**
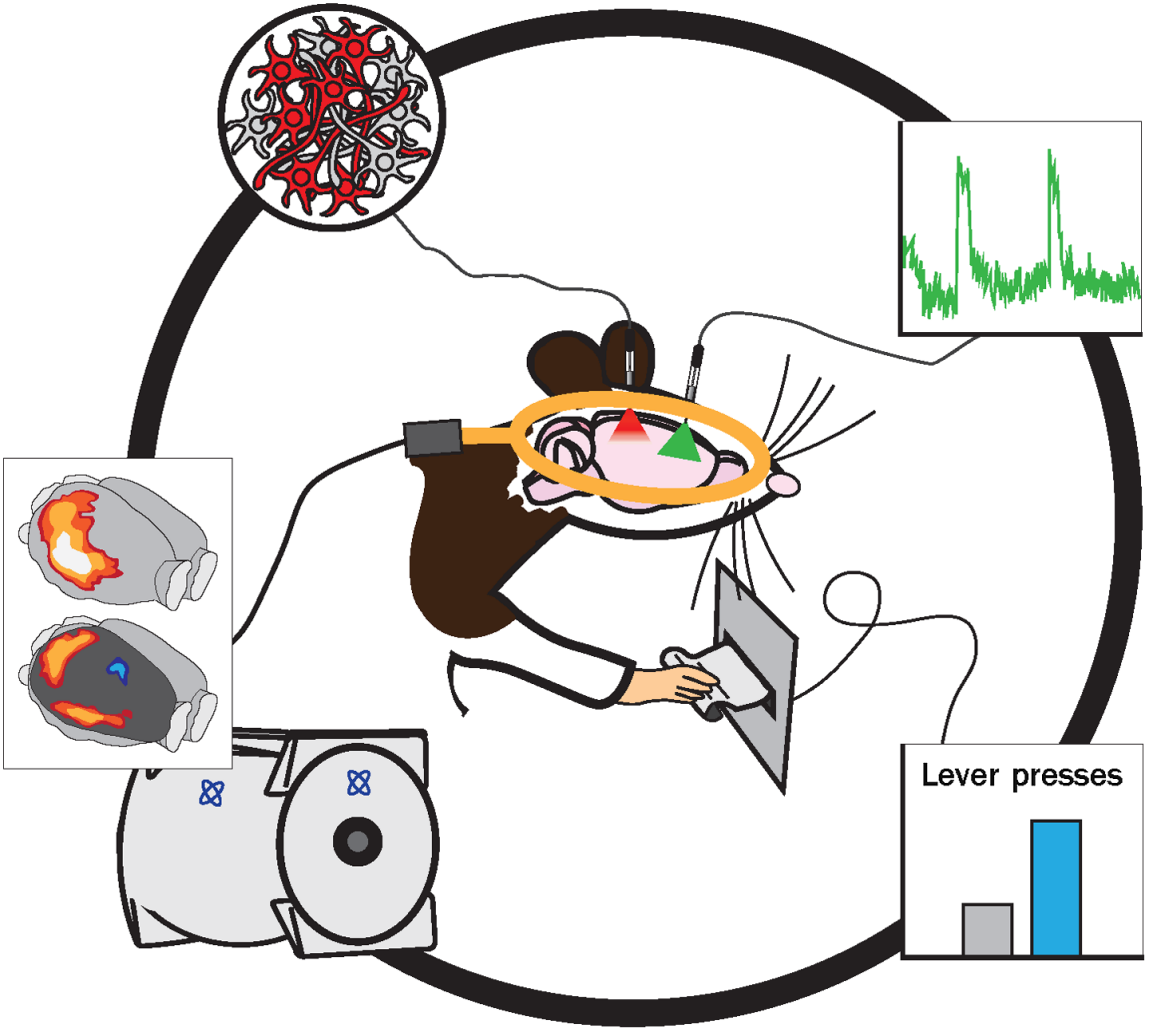
Connecting the dots between cell populations, whole-brain activity, and behavior. Opto-fMRI allows for the unique opportunity to genetically and optically manipulate targeted brain regions, projections, or cell types [shown as an implanted optical fiber and (red) light source in the rat brain and represented by targeted (red) neurons] and simultaneously (1) record ${\mathrm{Ca}}^{2+}$ activity in a region-, projection-, or cell-type-specific manner [shown as an implanted optical fiber and (green) light source and representative ${\mathrm{Ca}}^{2+}$ time course (green)], (2) correlate behavioral output [shown as an operant lever press and representative bar graph (blue)] and/or (3) measure BOLD fMRI activity [shown as a surface coil over the rat’s brain (gold), MR machine and representative 3D-, whole-brain BOLD activity map] in rodent models.

Optogenetics and fMRI have each had tremendous impacts on science individually, and recent technological advances have allowed for the marriage of the two methods, providing valuable synergistic advantages to the fields of both neuroscience and neuroimaging. Previously, the use of electrical stimulation or electrophysiology during fMRI led to interference between electrical and magnetic signals and did not allow for a targeted manipulation of specific cell types or projections. However, optogenetic manipulations and optical recording of neural activity based on GCaMP signals are insensitive to MRI-related interference and thus are well suited to being conducted alongside fMRI. In addition, high cell-type specificity makes it possible to dissect the linkages of separate neuron populations to fMRI signals. Indeed, the replacement of electricity with light application has allowed for the manipulation of specific populations of cells and neural pathways^82^^–^^84^ and/or imaging of ${\mathrm{Ca}}^{2+}$ signaling^85^^,^^86^ in a chronic manner^87^ [Fig. 4(d)] during BOLD imaging, removing those technical disadvantages.^88^ In addition, opto-fMRI offers the ability to simultaneously manipulate activity in individual circuits with a readout of whole-brain changes, with the option to examine the corresponding impacts on behavior or answer fundamental questions that lead to technological and analytical improvement in the neuroimaging field (Fig. 1).^34^^,^^87^^,^^89^^–^^101^ In the following sections, we cover recent advances and applications of opto-fMRI and future directions.

## 2 Recent Advances and Applications

### 2.1 Opto-fMRI: Actuators

#### 2.1.1 Anesthetized rodents

**Fig. 2:**
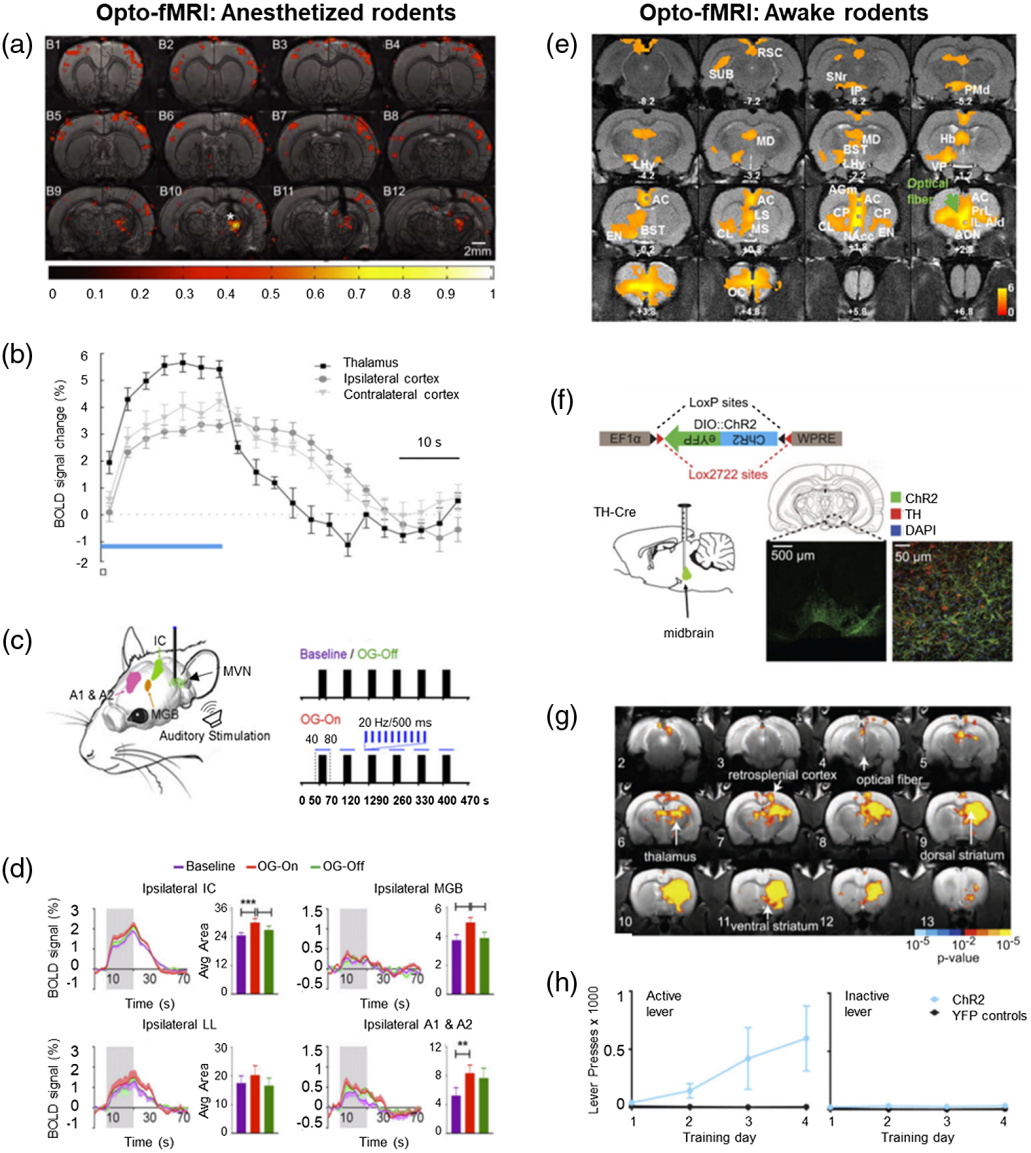
Concurrent fMRI and optogenetic manipulations in anesthetized and awake rodents. Revised from Refs. 133, 103, 34, 30. Representative experimental designs and results from opto-fMRI studies in (a)–(d) anesthetized and (e)–(h) awake rodents. (a) BOLD activation maps and (b) hemodynamic response functions in anesthetized rats receiving anterior thalamic optogenetic stimulation, illustrating BOLD responses ipsilaterally at the site of stimulation and both ipsilaterally and bilaterally in projection areas. Optogenetic stimulation can be presented alongside other sensory stimulations even in anesthetized rodents, as shown in (c) and (d). (c) Unilateral optogenetic stimulation was presented to the MVN (blue lines) concurrently with broadband noise stimulation (black bars). (d) The BOLD signal and averaged area under the signal profiles show that MVN optogenetic stimulation enhanced broadband noise-induced BOLD signaling in ipsilateral auditory pathway regions (IC, MGB, A1, A2). (e) BOLD activation maps that were averaged across different optogenetic stimulation paradigms targeting the IL in awake rats, showing activation patterns in both cortical and subcortical areas receiving projections from the IL. (f) Optogenetics allows for not only the targeted manipulation of a specific brain region but also cell-type specificity. By injecting a Cre-dependent ChR2 construct (green) into the midbrain of TH-Cre rats, light could be used to manipulate only the TH-expressing, or dopamine, cells in the midbrain. (g) BOLD activation maps during optogenetic stimulation of dopaminergic cells (red) in the midbrain in awake rats, showing robust, ipsilateral increases in activity in the dorsal and ventral striatum along with increases in other areas. BOLD activity during resting-state opto-fMRI can be correlated with behavior outside the scanner. (h) Active and inactive lever presses in rats receiving optogenetic stimulation of dopaminergic cells in the midbrain. Compared with control (YFP) rats (black line), ChR2-expressing rats (blue line) consistently had higher levels of active lever presses (compared with inactive lever presses) for midbrain light delivery, suggesting that the optogenetic stimulation pattern used during opto-fMRI is rewarding. Furthermore, there was a significant correlation between a rat’s preference for the active lever outside the scanner and the change in BOLD activity in the ventral striatum during optogenetic stimulation in the scanner. A1, primary auditory cortex; A2, secondary auditory cortex; AC, anterior cingulate cortex; AGm, medial agranular (frontal) cortex; AId, dorsal agranular insular cortex; AON, anterior olfactory nucleus; BST, bed nucleus of stria terminalis; CL, claustrum; EN, CP, caudate-putamen; endopiriform nucleus; Hb, habenula; IC, inferior colliculus; IL, infralimbic; IP, interpeduncular nucleus; LHy, lateral hypothalamus; LS, lateral septum; MBG, medial geniculate body; MD, mediodorsal nucleus; MS, medial septum; MVN, medial vestibular nucleus; NAcc, nucleus accumbens; OC, orbital cortex; PMd, dorsal premammillary nucleus; PrL, prelimbic; RSC, retrosplenial cortex; SNr, substantia nigra; SUB, subiculum; TH, tyrosine hydroxylase.

Since its first emergence,^88^ opto-fMRI applications have proven to be particularly useful for neurovascular coupling studies, allowing for direct examination and insights into the role of neural activity in the hemodynamic response. The addition of optogenetic manipulation to fMRI allows for the whole-brain visualization of the causal role of activating or inhibiting a specific cell type, defined by genetics, cell body location, or axonal projection target. As a crucial brain area for memory formation and retrieval, the hippocampus is an important target for the implementation of opto-fMRI. Light stimulation of the dentate gyrus (DG) in ChR2 transgenic rats led to an increase in the local BOLD response at both the site of stimulation and the CA3 area of the hippocampus as well as in projection areas, such as the primary somatosensory cortex (S1) and caudate putamen.^102^ Furthermore, there was an increased BOLD signal in the caudal DG, indicating that DG granule cells project to the longitudinal axis-level either through the mossy cells or direct projections.^102^ To investigate the role of excitatory neuronal activation in the generation of the BOLD signal, Lee et al.^88^ optogenetically activated CaMKIIα-expressing neurons in the neocortex or thalamus in anesthetized rats and found increased BOLD signals not only at the location of stimulation but also in brain areas that receive anatomical projections from the stimulated sites [Figs. 2(a) and 2(b)]. Similarly, optogenetic activation of pyramidal neurons in the S1 of ChR2 transgenic mice led to a positive BOLD response at the stimulation site, and optical pulse trains led to positive BOLD responses in projection areas in a consistent and repeatable manner.^98^ Furthermore, pulse train-generated BOLD activity correlated with single-unit, multi-unit, and local field potential activity.^98^ Iordanova et al.^94^ demonstrated related results in rats by optogenetically activating glutamatergic neurons in the forelimb somatosensory cortex during BOLD fMRI. They found that the hemodynamic response is consistent with that seen during sensory stimuli, showing activation, again, at the site of stimulation as well as the projected regions, including the primary motor cortex (M1), secondary somatosensory cortex (S2), striatum, and thalamus.^94^ In a corresponding study, Scott and Murphy^89^ optogenetically activated deep layer pyramidal neurons in the sensorimotor cortex or applied stimulation to the forepaw and measured global blood flow changes. Pharmacological blockade of glutamatergic signaling in the sensorimotor cortex significantly reduced forepaw stimulation-evoked hemodynamic response but had no effect on optogenetically evoked hemodynamic response.^89^ These results suggest that local glutamatergic signaling differentially regulates neurovascular coupling, depending on the type of stimulus applied. Finally, optogenetic stimulation of excitatory neurons in the medial vestibular nucleus (MVN) of rats led to robust fMRI activation in bilateral sensorimotor and high-order cortices, thalamus, and hippocampus.^103^ Furthermore, auditory and visual stimuli presented concurrently with optogenetic activation of MVN led to enhanced positive fMRI signals, notably in the auditory cortex and various regions in the ipsilateral visual pathway, suggesting an enhanced response to sensory stimuli.^103^ These results provide evidence that MVN activation regulates brain-wide activity and the response to sensory stimuli [Figs. 2(c) and 2(d)].^103^ Together, these studies demonstrate the utility of opto-fMRI in elucidating the local and global impact of the targeted manipulation of a single brain area.

Given its versatility, opto-fMRI offers a unique opportunity to elucidate specific roles of individual brain regions during different conditions. Some opto-fMRI studies have shown that fMRI response can vary in hemisphere laterality and direction (positive or negative), depending on optogenetic protocol, providing further support for the nuanced role of regional brain activity in global brain functioning. Lebhardt et al.^104^ unilaterally applied optogenetic stimulation to the hippocampus of CaMKIIα-Cre mice and found that glutamatergic neuron activity in the hippocampus (dependent on where along the dorso-ventral axis) led to a significant positive BOLD response, bilaterally at the site of stimulation, frontal lobe, and septum; ipsilaterally in the nucleus accumbens (NAc); and contralaterally in the striatum. Opto-fMRI of the ventrolateral orbitofrontal cortex (VLO) showed that this area also exhibits a global influence on brain activity.^105^ Specifically, optogenetically driving excitatory thalamic projections to the VLO led to opposing effects, depending on frequency of stimulations, and these effects were mediated by GABA signaling and activity in the zona incerta.^105^ Low-frequency stimulations led to widespread, bilateral decreases in brain activity, whereas high-frequency stimulations led to widespread, ipsilateral increases in brain activity.^105^ These studies not only underline the importance of careful consideration when planning optogenetic administration protocols but also highlight the dynamic and multifaceted role of individual brain regions, cell populations, and projections.

An increasing number of studies have shown that opto-fMRI can be beneficial when attempting to directly evaluate causal estimation methods in fMRI data. For instance, to biologically validate a multivariate dynamical systems (MDS) state-space model, optogenetic stimulation of M1 was utilized to elicit an increased hemodynamic response in the downstream regions of the thalamus and caudate putamen. These results supported the hypothesis that MDS state-space models can accurately and reliably estimate causal interactions of fMRI data.^99^ Furthermore, dynamic causal modeling (DCM) was also shown to accurately reproduce results from a study in which D1- or D2-expressing dopamine neurons in the striatum were optogenetically activated during fMRI, with the strongest model connections mimicking those of optogenetically stimulated pathways.^106^ The authors suggest that the use of DCM could facilitate the future design of more effective optogenetic neuromodulation therapies.^106^ Finally, using a modeling method that takes into account the scattering and absorption of light in the brain tissue and the relative density of regional opsin expression during opto-fMRI, Christie et al.^107^ were able to provide evidence for direct proportionality between the volume of neural tissue activated by optogenetics and the BOLD signal in the cerebral cortex. These results suggest that computational models could be a critical tool for future opto-fMRI studies.

Even in anesthetized rodents, opto-fMRI can be utilized to examine the network-level mechanisms that underlie the role of specific brain regions in certain brain/behavioral states. Liu et al.^108^ optogenetically stimulated the rat central thalamus during fMRI and found widespread activation of the forebrain and transition to a state of arousal, in a frequency-dependent manner. Opto-fMRI has also been utilized to study the role of the hippocampus in rodent seizure activity. In rats, frequency-dependent optogenetic stimulation in subregions of the hippocampus led to differential changes in whole-brain BOLD activity. Importantly, high-frequency stimulations in the intermediate hippocampus predicted behavioral seizures, and this effect involved BOLD activity in the DG.^105^ Furthermore, using opto-fMRI to study the initiation and termination of seizures from dorsal or ventral hippocampus in rats, Duffy et al.^109^ demonstrated distinct profiles of afterdischarges from differing hippocampal areas and provided insight into how seizures could be inhibited. Lee et al.^110^ used D1- and D2-Cre mice to optogenetically activate distinct dopamine populations and found that activation of either inhibitory population led to positive BOLD signals in the striatum, as well as opposing responses in projection areas, such as the thalamus, globus pallidus, and substantia nigra, providing evidence for the differential influence of these two striatal populations in whole-brain BOLD activity. These results were further supported by the confirmation of canonical control of locomotor behavior by optogenetic stimulations of D1- and D2-medium spiny neurons in animals while awake.^110^ Optogenetic activation of ventral tegmental area (VTA) dopamine neurons in TH::Cre rats enhanced intracranial self-stimulation (ICSS), a measure of reinforcement, in awake animals and, in anesthetized animals, led to an increase in BOLD and CBV in areas that receive innervations from the VTA, notably the NAc. Surprisingly, however, hemodynamic signals also increased in areas that receive little or no anatomical innervations from the VTA, such as dorsal striatum and globus pallidus, suggesting that mesolimbic and nonlimbic basal ganglia circuits are, instead, functionally connected and that they play a role in reward-related behavior.^111^ Brocka et al.^96^ further elucidated the role of the VTA in global brain activity using multiple approaches: (1) selective optogenetic activation of VTA dopamine neurons using TH::Cre rats, (2) joint optogenetic activation of VTA dopaminergic and glutamatergic neurons, and (3) nonspecific VTA neuron activation using electrical stimulation. Both optogenetic protocols led to increased ICSS, further validating the role of VTA dopamine and glutamate in reward-related behaviors. However, the BOLD signal patterns differed greatly between groups, with results supporting the role of mainly nondopaminergic (perhaps mostly glutamatergic) VTA signaling in brain-wide BOLD responses.^96^ Finally, optogenetic activation of serotonergic activity in the dorsal raphe nucleus (DRN) of ePet-Cre mice increased the hemodynamic response in the region of stimulation but mostly led to decreased CBV in projection areas and suppression of cortical delta oscillations.^97^ In fact, postsynaptic expression of serotonin (5HT) receptors was a much better predictor of DRN 5HT functional connectivity than previously established anatomical connections. Furthermore, acute stress led to a circuit-wide dampening of DRN output, and fluoxetine opposed this effect, leading to an enhancement of DRN functional connectivity.^97^ Collectively, these studies show that opto-fMRI can serve as a tool to test hypotheses regarding neuroanatomical pathways and their canonical as well as novel roles in behavior in a global, whole-brain manner.

#### 2.1.2 Awake rodents

The development of awake imaging in rodents^18^^,^^112^^–^^119^ has opened the door for a more translational integration of other neuroscience techniques, such as optogenetics. Opto-fMRI allows for the expansion of knowledge of a brain region and its whole-brain network from strictly anatomical to more of a functional understanding in awake and behaving rodents. Through the optogenetic activation of the medial prefrontal cortex (mPFC) during fMRI in awake rats, Liang et al.^34^ demonstrated a functional network of the mPFC that includes prefrontal, striatal, and limbic regions of the brain [Fig. 2(e)]. The increases in BOLD signal in these areas were robust, reproducible, and further supported by electrophysiological recordings.^34^ In addition, BOLD signals were substantially reduced when animals were anesthetized.^34^ Similarly, Desai et al.^120^ optogenetically stimulated the somatosensory cortex and analyzed how the cortical and subcortical targets of pyramidal cells are recruited in awake versus anesthetized rodents. In both groups, there was a substantial positive BOLD response in multiple brain areas, including functionally related and unrelated cortical and striatal regions.^120^ Furthermore, the awake group displayed a significant increase in BOLD activity at the region of optogenetic activation and downstream regions, as well as increased temporal correlations between pairs of regions, indicating stronger functional connectivity, compared with the anesthetized group.^120^ Collectively, these studies show that evoked hemodynamic responses display stronger BOLD activation in awake versus anesthetized rodents, further highlighting the value of awake rodent opto-fMRI studies.

Although neuroimaging studies have primarily focused on the relationship between hemodynamic responses and neuronal activity, some have suggested a role for glia in the control of BOLD signaling. Opto-fMRI studies provide an ideal tool to approach these hypotheses. Using transgenic mice with step-function opsin-type ChR2 expressed in either neurons or astrocytes, Takata et al.^121^ transcranially illuminated the cortex to specifically activate either neurons or astrocytes and showed an increased, repeatable BOLD response in the cortex of both groups. In addition, astrocyte-driven BOLD activity accompanied oxygen consumption without the modulation of neuronal activity, and this effect was shown to be associated with the synthesis of acetyl-carnitine via oxidative glucose metabolism at the site of the BOLD signal.^121^ These results were further supported with electrophysiological recordings and histochemical markers for cellular activation.^121^ Collectively, these data indicate that astrocyte activation alone is able to evoke a BOLD response through different mechanisms than neuronal activation, which is a very important consideration in future neuroimaging experimental designs and interpretations.

Two obvious benefits of utilizing opto-fMRI in awake rodents are (1) the ability to have a more accurate correlational value between brain and behavioral states and (2) the ability to perform real-time opto-fMRI during task-based imaging. For instance, Ferenczi et al.^30^ utilized opto-fMRI to examine the twofold nature of reward by looking at both the role of midbrain dopaminergic activation in global BOLD activity patterns and reward-related behavioral responses and the mPFC’s control of interactions between subcortical regions and their subsequent role in hedonic behavioral responses. Unilateral optogenetic stimulation of the midbrain led to robust, ipsilateral BOLD activity increases in the dorsal and ventral striatum, as well as in other brain regions, such as retrosplenial cortex and thalamus [Figs. 2(f) and 2(g)].^30^ Furthermore, lever pressing increased over training days in animals expressing ChR2, indicating that stimulation of dopaminergic midbrain cells is rewarding [Fig. 2(h)].^30^ On the other hand, by optogenetically activating mPFC, they found a reduction in midbrain dopamine-driven striatal activity that also resulted in an inhibition of dopaminergic ICSS.^30^ Furthermore, chronic optogenetic activation of mPFC led to specific brain-wide functional interactions between mPFC, orbital cortex, and ventral striatum and decreased natural-reward behaviors, indicating induction of anhedonia.^30^ Together, these results shed light on the role of mPFC in the orchestration of interactions between spatially distant brain regions and the expression of reward-related behaviors. In conclusion, studies thus far have focused on rs-fMRI and its correlation with behavioral outcomes, but the utilization of this technique during task-based imaging will surely be popular in years to come.

### 2.2 Opto-fMRI: Indicators

#### 2.2.1 Anesthetized rodents

**Fig. 3:**
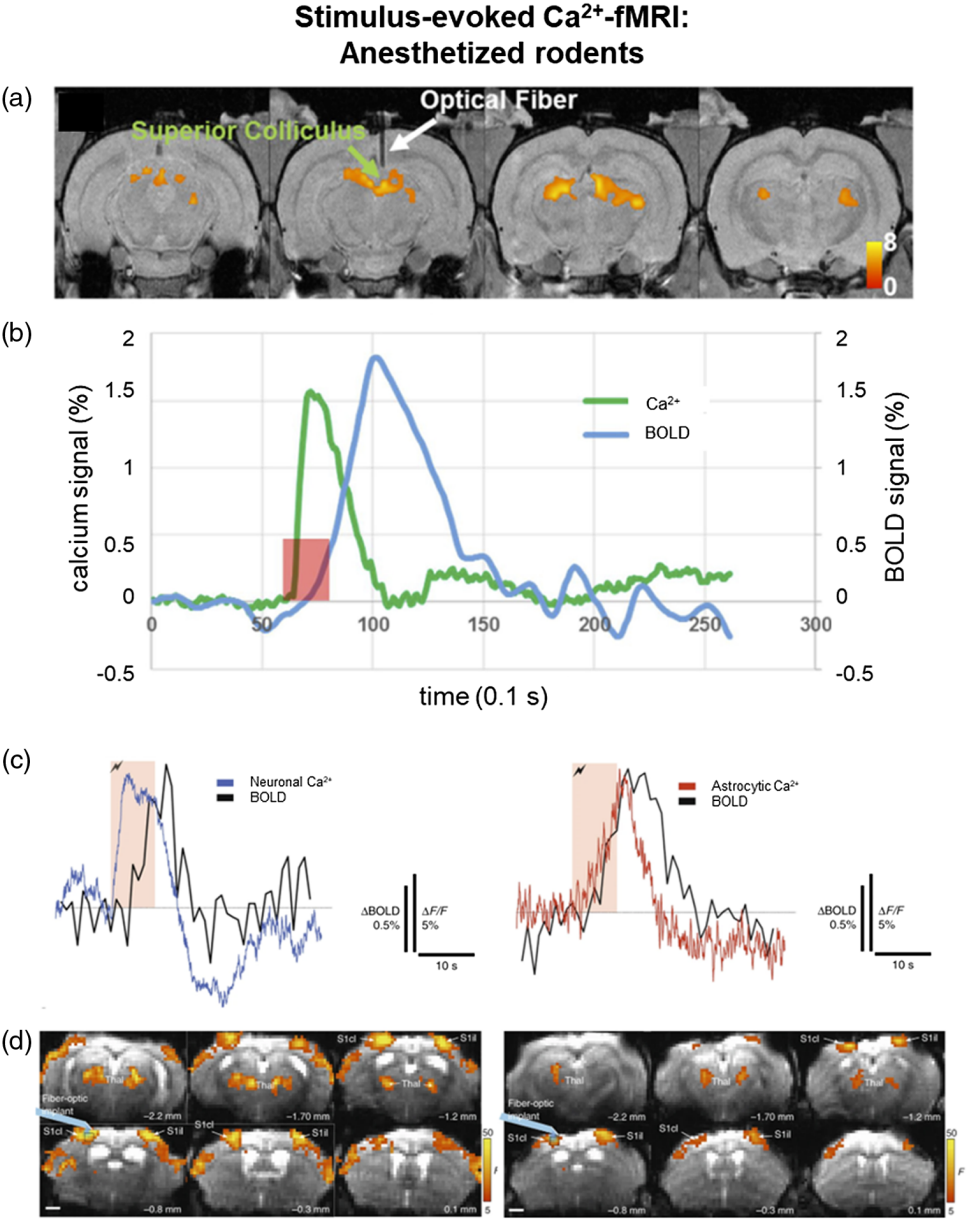
Correlations between concurrent stimulus-evoked fMRI BOLD and optogenetic calcium activation signals in anesthetized rodents. Revised from Refs. 122 and 87. (a) BOLD activation maps during visual stimulation in anesthetized rats, from the first published concurrent fMRI and calcium fiber photometry study. (b) Time courses of BOLD (blue) and calcium (green) signals in the superior colliculus during visual stimulation, illustrating the synchronization of the increase in calcium activity and visual stimulation paradigm and the difference in timing between calcium activity and BOLD activity, due to the hemodynamic decay. (c), (d) Through the use of neuron- or astrocyte-specific GCaMP expression, the role of the different types of cells in the brain in stimulus-induced changes in the BOLD signal can be distinguished. (c) Averaged time courses of BOLD (black) and neuronal calcium (blue on left) or astrocytic calcium (red on right) in primary somatosensory cortex during electrical hindpaw stimulation. (d) Significant statistical activation maps of correlations between BOLD and calcium signals during hindpaw stimulation in mice expressing either neuron-specific (left) or astrocyte-specific (right) GCaMP6. In these maps, a voxelwise generalized linear model analysis using the stimulus-evoked calcium signal trace was convolved with the hemodynamic response function as the regressor. S1cl, hind-limb area of the primary sensory cortex contralateral to the stimulated hind paw; S1il, hind-limb area of the primary sensory cortex ipsilateral to the stimulated hind paw; Thal, thalamus.

Since its first published application, the combination of GCaMP-based fiber photometry and fMRI has proven to be a valuable tool for examining the neural basis of BOLD signaling in both rats^122^^,^^123^ and mice.^87^ Reference 122 was the first study to demonstrate the feasibility of simultaneously and repeatedly measuring GCaMP and BOLD signaling in anesthetized rodents, showing that visual stimulation significantly increased ${\mathrm{Ca}}^{2+}$ and BOLD activity in the superior colliculus in a reproducible manner [Figs. 3(a) and 3(b)]. This technique was further used to show that ${\mathrm{Ca}}^{2+}$ slow-oscillation wave activity in S1 correlated with cortex-wide BOLD activity.^123^ This study was able to establish a direct connection of neurophysiologically defined slow oscillations to macroscopic fMRI signaling, suggesting that the entire cortex is involved in these so-called local “slow wave events.”^123^ He et al.^91^ further expanded on this multimodal technique by developing a method to detect vessel-specific rs-fMRI through a combination of BOLD and CBV imaging and ${\mathrm{Ca}}^{2+}$ recordings. By separating out arteriole- (CBV) and venule- (BOLD) sensitive fMRI measurements and concurrently measuring fluctuations in GCaMP signaling from neighboring neurons, they showed that opto-fMRI can resolve functional connectivity at the level of individual vessels.^91^ Pais-Roldán et al.^93^ created an fMRI index of brain state arousal by monitoring pupil dilations during combined GCaMP fiber photometry and fMRI. In anesthetized rodents, the global fMRI signal oscillated similarly to ${\mathrm{Ca}}^{2+}$ signaling, in a way that seemed to be linked to arousal;^93^ further elucidating these fluctuations could be useful for a better understanding of neurovascular coupling and results from future studies.

**Fig. 4:**
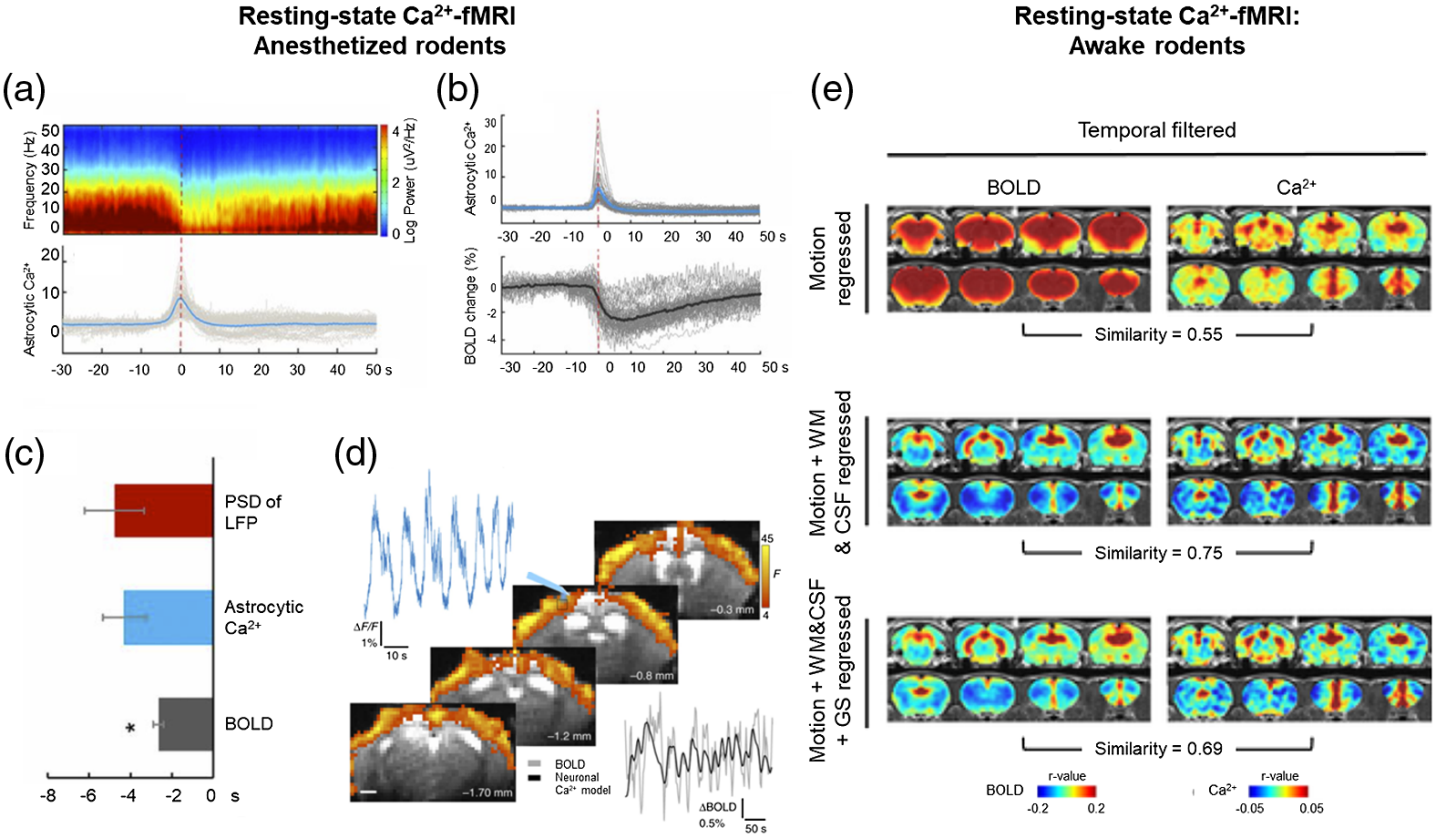
Correlations between concurrent resting-state fMRI BOLD and optogenetic calcium activation signals in anesthetized and awake rodents. Revised from Refs. 92; 87; 126. Representative results from concurrent calcium and resting-state fMRI studies in (a)–(d) anesthetized and (e) awake rodents. (a) Top panel: Averaged spectrogram of the LFP, bottom panel: resting-state astrocytic calcium signal, with a negative correlation between the peak spike time and the LFP (red dashed line). (b) Top panel: Resting-state astrocytic calcium signal, bottom panel: averaged time course of resting-state BOLD signal, with a negative correlation between the peak calcium spike time and BOLD (red dashed line). (c) Estimated onset times of the LFP PSD (red), resting-state astrocytic calcium spikes (blue) and BOLD signal reduction (gray). Together, (a)–(c) illustrate that astrocyte-specific GCaMP expression can contribute to the understanding of the role in the resting-state BOLD signal. Specifically, these results show that intrinsic calcium spikes in the cortex negatively correlated with neuronal and BOLD signals. (d) Example time course of neuron-specific calcium signal (blue), significant statistical activation map of correlation between BOLD and neuron-specific calcium signal, and averaged time course of BOLD (gray) and a predicted model based on neuronal calcium signaling (black) in primary somatosensory cortex during resting-state in rodents 6 months after optical fiber implantation. This illustrates the ability to chronically perform calcium-fMRI studies. (e) Calcium-fMRI information can also be used to inform upon processing of BOLD activity data. Comparisons of BOLD activity maps and calcium-signal derived dorsal hippocampus maps (used as the ground truth) using different iterations of common preprocessing techniques. LFP, local field potential; PSD, spectral power shift. (a)–(c) Copyright National Academy of Sciences 2018.

Similar to opto-fMRI, studies using GCaMP-fMRI can be useful for investigating not only neuronal activity, as demonstrated above, but also the role of astrocytes in neurovascular coupling events. For instance, in a methods paper, Schlegel et al.^87^ showed differential electrical paw stimulation-evoked BOLD activation patterns when signaling was correlated with astrocyte-specific versus neuron-specific GCaMP6 activity [Figs. 3(c) and 3(d)]. In addition, Wang et al.^92^ showed that astrocytic ${\mathrm{Ca}}^{2+}$ activity in the rat brain is bidirectionally correlated with the BOLD signal. In particular, this study showed that a conventionally evoked BOLD fMRI signal positively correlated with ${\mathrm{Ca}}^{2+}$ activity in thalamic astrocytes, whereas intrinsic ${\mathrm{Ca}}^{2+}$ spikes in the thalamus were negatively correlated with both local field potentials and the cortical BOLD signal^92^ [Figs. 4(a)–4(c)]. Future studies involving GCaMP-fMRI in both neurons and astrocytes will be important for a more nuanced understanding of neurovascular coupling.

To more easily pair with fMRI, most GCaMP applications thus far have been with fiber photometry-based monitoring in an isolated brain region. However, optical setups that utilize concurrent multiple-site ${\mathrm{Ca}}^{2+}$ imaging and fMRI have recently been developed, allowing for a true whole-brain monitoring approach. Tong et al.^124^ demonstrated the capabilities of a camera-based, dual channel ${\mathrm{Ca}}^{2+}$ fiber photometry and fMRI approach by measuring GCaMP signaling in the superior colliculus and lateral geniculate nucleus while obtaining rs-fMRI or task-evoked fMRI in rats. Interestingly, they found a robust correlation between BOLD and GCaMP signals in both areas during a visual stimulus presentation and less correlation during the resting state, suggesting a differential role of ${\mathrm{Ca}}^{2+}$ signaling in neurovascular coupling during task-evoked and rs-fMRI.^124^ In addition, using a modified and fMRI-compatible fiber bundle approach in GCaMP6f mice, Lake et al.^125^ were able to simultaneously capture spontaneous BOLD and ${\mathrm{Ca}}^{2+}$ activity from almost the entire cortex with improved spatial resolution. Importantly, they observed both correlated trial-to-trial variations and correlated spontaneous activity between ${\mathrm{Ca}}^{2+}$ and BOLD signaling, highlighting the importance of examining spontaneous whole-brain activity.^125^ Technical advances such as these and those highlighted previously continue to open up the possibilities for opto-fMRI applications in the neuroscience and neuroimaging fields.

#### 2.2.2 Awake rodents

To our knowledge, the first published account of concurrent measurements of fiber photometry-based ${\mathrm{Ca}}^{2+}$ signaling and BOLD fMRI in awake rodents is in Ref. 100. In this study, a single-echo planar imaging (EPI) sequence was used to correlate rs-fMRI BOLD activity with GCaMP signaling to characterize the role of the hippocampus in global brain activity. In addition, a multiecho EPI sequence was used to differentiate neural and non-neural components of the global rs-fMRI signal. The combined results from both methods provide twofold support for a neural basis of the global brain signal in awake rodents, possibly involving the hippocampo- and thalamo- cortical networks. To examine the relationship between neuronal spiking activity (measured by GCaMP) and BOLD signaling during the resting state, Ma et al.^126^ utilized concurrent ${\mathrm{Ca}}^{2+}$-based fiber photometry and rs-fMRI in rodents. They showed robust couplings between ${\mathrm{Ca}}^{2+}$ and BOLD activity in areas of the default mode network, such as dorsal hippocampus, providing further support for the role of neuronal spiking in the rs-fMRI BOLD signal.^126^ Importantly, using ${\mathrm{Ca}}^{2+}$ signaling as the ground truth, they also showed that common data preprocessing techniques differentially impact functional connectivity mapping^126^ [Fig. 4(e)]. In a task-based fMRI study, Zhang et al.^127^ measured BOLD responses during light onset and offset for the first time in awake rodents. They developed a high-dimension linear model that allowed for the removal of ongoing, highly correlated, between-region BOLD signaling, which led to a reduction in cross-trial variability, an ongoing issue in fMRI processing and analyses.^127^ Furthermore, through the employment of concurrent ${\mathrm{Ca}}^{2+}$-based fiber photometry, they showed that this removal increased the BOLD activity coherence with underlying GCaMP activity.^127^ Most importantly, these recent articles highlight how consequential using opto-fMRI in awake rodents can be to better interpret results from both resting-state and task-based fMRI; the use of GCaMP-fMRI provides exciting opportunities for the future.

### 2.3 Opto-fMRI: Combined

In recent years, one group has built on previous technological advancements to develop a multimodal fMRI platform, combining optogenetic stimulation with concurrent fMRI and optical fiber ${\mathrm{Ca}}^{2+}$ recordings.^90^^,^^101^^,^^128^ This all-optical fMRI design provides a means to investigate detailed neurovascular coupling events in cortical and subcortical brain regions. Using a high spatial resolution, vessel-specific BOLD- and CBV-weighted fMRI mapping method, Chen et al.^128^ linked subcortical neural activity to the fMRI signal, while also measuring concurrent ${\mathrm{Ca}}^{2+}$ events in anesthetized rats. They described differences in optogenetically evoked and spreading depression-like ${\mathrm{Ca}}^{2+}$ events and highlighted the role of the CA1 hippocampal region in seizure activity.^128^ With an MRI-guided robotic arm to better target small, subcortical brain areas during stereotaxic surgeries,^101^ this multimodal fMRI platform was also used to optogenetically modulate the corpus callosum to (1) identify both orthodromic and antidromic signaling effects on global network activity and (2) map the temporal characteristics of corpus callosum-mediated thalamocortical activation in the barrel cortex.^90^ Furthermore, they characterized the correlations between fMRI and ${\mathrm{Ca}}^{2+}$ activity during both optogenetic and whisker stimulation, showing an inhibition of contralateral barrel cortex BOLD and ${\mathrm{Ca}}^{2+}$ signaling.^90^ These studies illustrate the ability of multimodal fMRI to characterize local neurovascular coupling events at the single-vessel resolution and circuit- or population-specific manipulation effects on global network activity *in vivo*. In addition, in a promising methodological paper, Desjardins et al.^129^ demonstrated the feasibility of longitudinally combining optical stimulation, two-photon microscopy-based ${\mathrm{Ca}}^{2+}$ measurements and task-based fMRI in awake mice. They showed that, consistently in the same mice, BOLD fMRI data could be obtained in response to both optogenetic and sensory stimuli and that cranial windows did not significantly reduce the quality of fMRI data.^129^ As technical advancements progress, we as a scientific community are provided countless opportunities for future applications.

## 3 Potential Confounds and Important Considerations for Opto-fMRI

As with any technique, there are potential pitfalls that need to be considered and controls that should be implemented when utilizing both the individual tools and combinations of tools within opto-fMRI.

### 3.1 Specific to fMRI

As highlighted in previous sections, the majority of opto-fMRI studies thus far have been performed in anesthetized rodents. In addition to the variability in anesthetics used between different labs for fMRI studies, the influence of anesthesia in general is an important consideration when planning opto-fMRI studies. Briefly, anesthesia decreases the amplitude and increases the lag of the hemodynamic response, decreases baseline brain metabolism and temperature, alters cellular signaling, and affects all physiological processes that underlie the BOLD signal.^32^ For detailed reviews, see Refs. 32 and 130. Natural physiological factors should also be considered as they can contribute to non-neural signal variation. These include respiratory and cardiac motion artifacts, individual variations in body temperature, and baseline differences in blood ${\mathrm{CO}}_{2}$ and $O_{2}$, blood pressure, CBV, and CBF (reviewed in Refs. 131 and 132). Other considerations within neuroimaging experiments include the effects of species, sample size, sex, and animal preparation (reviewed in Ref. 130). For example, Mandino et al.^130^ reported a bias toward male animal fMRI studies (54% versus 22% female-inclusive studies); however, a more surprising part of their report is that 22% of animal fMRI studies completely omit the sex. Out of the 36 opto-fMRI papers reported in the current review, 22% also completely omit the sex (see Table 1). By doing so, reproducibility is hindered, and the possible contributions of sex to neurovascular coupling are ignored. Furthermore, Jonckers et al.^136^ provided evidence that functional networks extracted using independent component analysis (ICA) were more unilateral in mice than they were in rats, highlighting the importance in species considerations during experimental design and conclusions. The addition of disease model-dependent effects and behavioral-induced changes to BOLD signaling and functional networks are also relevant when planning experimental designs and controls. For instance, behavioral assays involving reward-cue associations can reduce functional connectivity between nodes in the rodent default mode network,^137^ and unconditioned fear stimuli can lead to reductions in functional connectivity after exposure.^31^ Finally, Mandino et al.^130^ pointed out that, although fMRI is invaluable, there is a lack of standardization, particularly in rodent fMRI, and this phenomenon makes it difficult to achieve reproducibility and reach broad conclusions in the neuroimaging field. This is often due to differences in experimental design, data processing, and data analyses, and this will further be a confound when combining other techniques with fMRI. According to Pan et al.,^138^ the choice of anesthesia administration and preprocessing pipeline methods have the largest contribution and should perhaps be a focus for improvement in future opto-fMRI studies.

**Table 1:** Overview of opto-fMRI studies included in this review article. The first column specifies wild-type and transgenic mouse and rat strains. The second column specifies the transgene or promotor of the transgenic rodent strains, and the third column states whether male or female rodents were utilized. The following three columns indicate which technique was included in the study: optogenetics, GCaMP, fMRI or a combination of two or three techniques. The seventh column includes whether animals were anesthetized or awake, and the last column is the citation of the study. ? not stated; F, female; LE, Long Evans rat; M, male; SD, Sprague Dawley rat; SSFO, step-function opsin; TH, tyrosine hydroxylase.

Animal strain	Transgene/promoter	Sex	Optogenetics	GCaMP	fMRI	Awake or anesthetized	Citation
SD	—	M	X	X	X	Anesthetized	Ref. 101
SD	—	M	X	X	X	Anesthetized	Ref. 90
SD	—	M	X	X	X	Anesthetized	Ref. 128
C57BL/6	VGAT-ChR2, Emx1-Cre/Ai32	M, F	X	Cranial windows	X	Awake	Ref. 129
SD, Fisher	—	F	X	—	X	Anesthetized	Ref. 133
C57BL/6	ChR2	?	X	—	X	Anesthetized	Ref. 98
SD	—	F	X	—	X	Anesthetized	Ref. 108
SD	—	M	X	—	X	Anesthetized	Ref. 94
SD	—	M	X	—	X	Anesthetized	Ref. 105
SD	—	M, F	X	—	X	Anesthetized	Ref. 99
C57BL/6NCrl	CaMKIIα-Cre	M	X	—	X	Anesthetized	Ref. 104
BAC-Cre mouse	Drd1a-262, Drd2-44	M	X	—	X	Anesthetized	Ref. 110
LE	TH::Cre	M	X	—	X	Anesthetized	Ref. 111
BAC-Cre	Drd1a-262 (D1), Drd2-44 (D2)	M	X	—	X	Anesthetized	Ref. 106
SD	—	M	X	—	X	Anesthetized	Ref. 107
LE	TH::Cre	?	X	—	X	Anesthetized	Ref. 96
SD	—	F	X	—	X	Anesthetized	Ref. 134
Tg (Fev-cre) 1Esd mouse	ePet-CRE	?	X	—	X	Anesthetized	Ref. 97
SD	—	M	X	—	X	Anesthetized	Ref. 103
SD	—	M	X	—	X	Anesthetized	Ref. 109
W-TChR2V4 rat	ChR2-venus	?	X	—	X	Anesthetized	Ref. 102
LE	—	M	X	—	X	Awake	Ref. 34
LE	TH::Cre	F	X	—	X	Awake	Ref. 30
tetO-SSFO-ChR2 mouse	Chrm4 (neuron), Mlc1 (astrocyte)	?	X	—	X	Awake	Ref. 121
C57BL/6	ChR2	?	X	—	X	Awake, anesthetized	Ref. 120
C57BL/6	ChR2	?	X	—	X	Anesthetized	Ref. 89
LE—	—	M	—	X	X	Anesthetized	Ref. 122
Fisher	—	F	—	X	X	Anesthetized	Ref. 123
SD	—	M	—	X	X	Anesthetized	Ref. 92
Unspecified rat	—	?	—	X	X	Anesthetized	Ref. 91
SD	—	M	—	X	X	Anesthetized	Ref. 124
SD	—	?	—	X	X	Anesthetized	Ref. 93
Slc17a7-Cre; Camk2a-tTA; Ai93 mouse	GCaMP6f	F, M	—	X	X	Anesthetized	Ref. 125
LE—	—	M	—	X	X	Awake	Ref. 135
LE	—	M	—	X	X	Awake	Ref. 126
LE	—	M	—	X	X	Awake	Ref. 127

### 3.2 Specific to Optogenetics

When conducting opto-fMRI experiments, the influence of opsin gene and light source should be considered, and as always, careful controls need to be put in place. The light source for opto-fMRI can lead to heating artifacts, tissue damage, and nonspecific effects.^139^^–^^143^ One obvious and effective way to control for these possibilities is to include a control group of animals with null (i.e., no opsin) viral vector injections but that receive light application. Inclusion of histological and/or electrophysiological recordings can also assist in two ways: (1) with the verification of cell health following long-term expression of opsins and optogenetic stimulation/inhibition and (2) with the verification of specificity of opsin expression within a brain area, cell population, and/or pathway. Optic fiber implantation for light delivery and/or ${\mathrm{Ca}}^{2+}$ imaging also has the possibility of brain tissue damage and imaging artifacts; therefore, these effects should be taken into account during the preprocessing of fMRI data. A lighter, multimodal fiber system has been developed for ${\mathrm{Ca}}^{2+}$ fiber photometry to improve on this confound; however, the trade-off is that the probe is more prone to damage.^77^ Another important consideration is the stimulus pattern delivery. Previous studies have performed dose response curves of pulse patterns based on behavioral or physiological readouts to determine the desired protocol.^43^ Another option is to use electrophysiological results as a guide to match distinct activity patterns using light application.^144^ Finally, although GCaMP signaling can be measured using two-photon imaging and miniscopes, and these methods have single cell resolution, the majority of opto-fMRI applications use fiber photometry, which requires the imaging of bulk fluorescent signals (i.e., lower resolution).^74^^–^^77^ Therefore, if ${\mathrm{Ca}}^{2+}$ signaling is being recorded from a diverse population of cells, it is possible to lose nuanced details.

### 3.3 Combined

Technical considerations to be aware of when utilizing opto-fMRI include adjustments to imaging parameters, such as shimming and radio frequency power calibration, to account for optic fiber implantation.^145^^,^^146^ During optogenetic stimulation, the power settings of the light application need to be adequately balanced, being above a defined threshold intensity but not so intense that tissue heating leads to false BOLD signals. Strategies to account for this include performing a light dose response curve and including a nonopsin control group. Furthermore, the length of light pulses and their contribution to the BOLD signal need to be considered. For instance, Albers et al.^7^ pointed out that trains of 1-ms pulses could be the ideal protocol for fMRI measurements (see their review article for more information). In the special case of concurrent optogenetic stimulation and ${\mathrm{Ca}}^{2+}$ recordings in the same brain region during fMRI, high levels of fluorescence may saturate the detector, and a large intensity difference may obscure the fluorescence peak.^7^ Inclusion of artificially long “dead times”^147^ or subtraction of detector recovery from acquired data^142^ can help prevent these issues.^7^ In addition, separation of wavelengths for the opsin and ${\mathrm{Ca}}^{2+}$ indicators, such as through the use of redshifted opsins, and spatially separating excitation and detection circumvent imaging interference and artifacts.^7^ Due to the quickly accelerating popularity of ${\mathrm{Ca}}^{2+}$ imaging, the problem of lack of standardization has arisen in the application of this technique as well.^70^ The substantial amount of data generated, particularly with the use of two-photon imaging and miniscopes, further contributes to this confound. One must also take into account the additional data resulting from the combination with fMRI. A few groups are attempting to find solutions to help with data management, including the use of more automated methods, such as ICA^148^ and machine learning techniques.^149^ Choosing the most scientifically sound way to process and analyze concurrent fMRI and optogenetic data will continue to be an important consideration in studies applying these techniques.

## 4 Future Directions

### 4.1 fMRI and Optogenetics in Spinal Cord and Peripheral Nervous System

Optogenetics is oftentimes superior to therapeutic electrical stimulation due to its cell-type specificity, allowing for direct treatment of underlying conditions without off-target effects and giving it translational potential beyond the brain.^150^ Despite numerous characteristics that impede the traditional use of optogenetics in the spinal cord and peripheral nervous system (PNS), such as tissue complexity, cellular and molecular heterogeneity, flexibility, and immune responses, technical advances are making this application a reality (reviewed thoroughly in Ref. 151). Two hurdles in this process have been figuring out the best way to accommodate opsin expression and to apply light delivery outside the brain. Using viral transduction, viral vectors containing opsins have successfully been injected intraspinally, intratumorally, and intramuscularly,^152^^–^^154^ and the feasibility of using grafts of opsin-expressing cells has been demonstrated for the modulation of muscle and glucose homeostasis.^155^^,^^156^ Light delivery has been administered in a variety of ways; however, transdermal illumination seems particularly promising, being applied to peripheral sensory afferents and smooth muscle.^1^^,^^157^ Optogenetic applications outside of the brain have been particularly successful for the control of somatosensation and pain, motor circuits, and even non-neural targets, such as muscle cells (see Ref. 151). For instance, both excitatory and inhibitory opsins were expressed specifically in nociceptors, allowing for bidirectional, transdermal light control over pain perception.^157^ Furthermore, optogenetic control of VGAT+ inhibitory interneurons in the dorsal spinal cord allowed for a thorough investigation of the role of these cells in locomotion and, in particular, mouse hindlimb circuits.^158^ As progress continues to be made through both the investigatory and therapeutic applications of optogenetics in the spinal cord and PNS, the addition of fMRI to monitor CNS response during such modulations could be beneficial. Opto-fMRI outside the brain could not only help validate therapeutic improvements but also help further elucidate the role of full brain-body connections on global brain function.

### 4.2 Clinical Applications of Opto-fMRI

Optogenetics and opto-fMRI can be used as a tool for investigating the neural mechanisms behind less understood therapeutic applications, such as transcranial magnetic stimulation and deep brain stimulation (DBS). DBS is currently being used to treat disorders such as Parkinson’s disease, epilepsy, chronic pain, major depression, and obsessive-compulsive disorder. However, the mechanism of action and effects on global brain functioning are still not well understood. In one example, Gradinaru et al.^82^ optogenetically dissected the neural mechanisms behind DBS treatment of Parkinson’s disease, showing that simply inhibiting glutamatergic cell bodies in the subthalamic nucleus was not sufficient to block Parkinson’s disease-related motor deficiencies and that it actually required a specific protocol being applied to afferent axons in the brain area. One group is using opto-fMRI to further elucidate the mechanisms underlying NAc DBS. Albaugh et al.^95^ optogenetically activated CamKIIα cells in the NAc and measured changes in the BOLD signal. Although more follow-up experiments need to be done, their results may suggest that NAc DBS results from antidromic activation of axons or fibers of passage.^95^ This finer tuned understanding of the mechanism of thalamic DBS treatment in Parkinson’s disease and NAc DBS would not be possible without optogenetics. In the future, combining other technologies, such as fMRI, with therapeutic optogenetics will no doubt prove useful when monitoring progress and further elucidating the mechanism of treatment.

In addition to being an excellent tool for dissecting cell type- and projection-specific mechanisms underlying other therapeutic tools, optogenetics has been suggested as a contender for treatment of various disorders.^159^ Under this goal, a lot of progress has been made in the preclinical space. For instance, in a rodent self-administration model of drug addiction, optogenetically induced reversal of cocaine-evoked neural plasticity in inputs from mPFC and ventral hippocampus to NAc D1R medium spiny neurons blocked cue-induced reinstatement up to 1 week after optogenetic application.^160^ Furthermore, through an exhaustive study to investigate the mechanism of thalamic neuromodulation in Parkinson’s disease, Gradinaru et al.^82^ uncovered a therapeutic target within the subthalamic nucleus. Using Thy1::ChR2 transgenic mice, in which ChR2 is abundant in afferent fibers but excluded from cell bodies, they showed that optogenetic high frequency stimulation of afferent fibers in the subthalamic nucleus robustly blocked motor symptoms that characterize Parkinson’s disease.^82^ In future studies, the addition of fMRI could shed more light on the mechanism and effectiveness of therapeutic optogenetics in a particularly translational nature.

As implied above, optogenetics can also be used therapeutically beyond the brain. In particular, optogenetic control has arisen as a contender for treating disorders including motor system dysfunction and spinal cord injury.^151^^,^^161^ A particularly exciting application was demonstrated by Alilain et al.^162^ They showed that optogenetic stimulation of spinal cord neurons near a paralysis-inducing diaphragm lesion restored independent breathing that lasted at least 24 h after light application ended.^162^ Paschon et al.^161^ cover various existing technological approaches that could be utilized in the treatment of spinal cord injury, including optogenetics. Among other approaches, they suggest ChR2-controlled neuronal differentiation following stem cell-based therapies, similar to what has been done in a rodent stroke model.^161^ Improvements in viral-mediated expression of opsins outside the brain and extension of light delivery capabilities will no doubt continue to allow for expansion of optogenetic therapeutic applications in the future. Although the majority of therapeutic applications of optogenetics have been in rodent models of human disorders, there have been recent improvements in the application of optogenetics in non-human primates,^163^ nudging us toward the ultimate goal of optogenetic treatment applications in humans. In fact, the first human application was recently published; optogenetics was used to partially restore vision in a patient with retinitis pigmentosa, a neurodegenerative disease that leads to blindness.^164^ In this patient, ChR2 was virally expressed in the ganglion of one eye, and light from the environment was transformed into a red wavelength representation using specialized goggles.^164^ Starting 8.5 months after opsin injection, the patient reported signs of visual improvement while using the goggles and showed an improvement in various visual and visuomotor tasks, compared with preoptogenetic treatment.^164^

## 5 Conclusions

Optogenetics, which includes both actuators (opsins) and indicators (sensors), and fMRI are two independently important neuroscience methods that, when combined, lead to valuable, synergistic benefits in the fields of neuroscience and neuroimaging. The technical advances that have allowed for opto-fMRI in anesthetized and awake rodents have led to invaluable applications, and the future possibilities are seemingly endless.
